# Canine *Dracunculus* Nematode Infection, Toledo, Spain

**DOI:** 10.3201/eid2608.201661

**Published:** 2020-08

**Authors:** Irina Diekmann, Alaa Aldin Alnassan, Majda Globokar, Nikola Pantchev, Lina Kurzrock, Leticia Hernandez, Javier Lopez, Ricardo Ruano, Silvia Herrero, Georg von Samson-Himmelstjerna, Jürgen Krücken

**Affiliations:** Freie Universität Berlin, Berlin, Germany (I. Diekmann, G. von Samson-Himmelstjerna, J. Krücken);; IDEXX Laboratories, Ludwigshburg, Germany (A.A. Alnassan, M. Globokar, N. Pantchev, L. Kurzrock);; IDEXX Laboratories, Barcelona, Spain (L. Hernandez, J. Lopez);; Clínica Mediterráneo, Madrid, Spain (R. Ruano, S. Herrero)

**Keywords:** dracunculiasis, dog, infection, mammalian, Spain, Europe, parasites, waterborne diseases, foodborne diseases, Guinea worm, nematodes, vector-borne infections, Dracunculus

## Abstract

A fragment of a *Dracunculus*-like worm was extracted from the hind limb of a 2-year-old dog from Toledo, Spain. Cytochrome oxidase I and rRNA sequences confirmed an autochthonous mammalian *Dracunculus* worm infection in Europe. Sequence analyses suggest close relation to a parasite obtained from a North American opossum.

The nematode genus *Dracunculus* contains 14 accepted species, 10 of which parasitize reptiles. The mammalian parasites include *D. fuelleborni*, *D. lutrae*, *D. insignis* and the human pathogen *D. medinensis*. Adult female *Dracunculus* worms are located in the host subcutaneous tissue, especially at the distal extremities (*1*). Human dracunculosis was an important neglected tropical disease, but successful eradication programs have eliminated the parasites from most endemic countries, with the exception of Chad, Ethiopia, South Sudan, Mali, and Angola. In 2019, only 53 human cases but 1,991 animal infections, predominantly in dogs, were reported worldwide (*2*). Infection occurs by ingestion of copepods containing third-stage larvae through drinking water or by feeding on paratenic hosts such as frogs, tadpoles, or fish (*3*,*4*). No genetic differences can be observed between worms infecting humans and dogs, which now appear to be important reservoir hosts (*5*). In addition, *D. lutrae* (host: North American river otter *Lontra canadensis*) and *D. insignis* (wide host range, including raccoons, several mustelids, and canids) worms infect predominantly carnivores in North America (*6*,*7*). 

The taxonomy of *Dracunculus* is based on morphologic characteristics of male worms, which are rarely available. Therefore, identification based on 18S rRNA has been used for phylogenetic analysis, and identification based on mitochondrial marker cytochrome oxidase C subunit I (COI) has been used for phylogeny, barcoding and intraspecific variation analyses (*6*).

## The Study

In summer 2018, a 2-year-old dog resembling a podenco (a local breed in Spain) was brought to a veterinarian in Madrid, Spain, with an oozing ulcer laterally in the tarsal region on the left hind limb. The dog had been in the owner’s possession for 1 year at the time. Before that, the dog lived on a pig farm in Toledo, Spain, where the previous owner did not take care of the animal and the dog had access to the food and water supplied for the pigs. Because of a case of abuse, the dog was rescued from the farm. 

**Figure 1:**
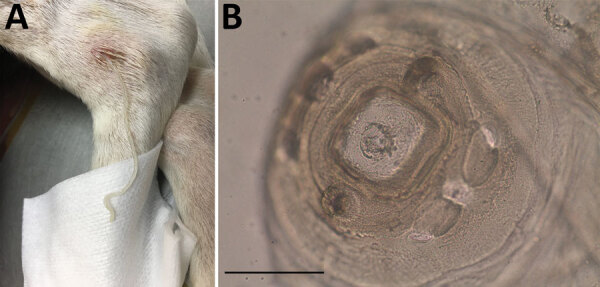
*Dracunculus* worm extracted from a dog in Toledo, Spain, 2018. A) Nematode extracted from the subcutaneous tissue of tarsal region on the left hind limb of naturally infected dog. B) Microphotograph of the anterior end with characteristic papillae. Scale bar represents 50 µm.

A 12-cm-long front end of a nematode was extracted from the ulcer (Figure 1, panel A). In the preceding year, the dog had been regularly dewormed, wore a collar to protect against fleas and ticks, and was treated with allopurinol against leishmaniosis; the animal had not traveled abroad. After the removal of the nematode fragment by the veterinarian, the nematode was fixed in 70% ethanol and submitted to IDEXX Laboratories (Ludwigshaven, Germany) for diagnosis. Morphologic examination of the nematode excluded a filarioid species, and a female *Dracunculus* worm was suspected on the basis of the structure of the papillae around the mouth capsule and the worm size (Figure 1, panel B) (*9*). For female specimens, species identification is impossible. No larval stages were found in the specimen because only the anterior end of the worm was collected and the uterus was probably lost.

We amplified and sequenced partial nuclear 18S and 28S rRNA and mitochondrial COI fragments (*10*–*12*; Appendix). From the aligned sequences, we constructed maximum-likelihood phylogenetic trees by using IQtree 1.6.12 (*13*). Partial 18S rRNA sequences (832 bp, GenBank accession no. MT311138.1) showed 99.6% identity to *D. lutrae* (GenBank accession no. JF934737.1), *Dracunculus* sp. V3104 (GenBank accession no. DQ503457.1), and *D. insignis* (GenBank accession no. AY947719.1) and 99.5% identity to *D. medinensis* (GenBank accession no. MK881307.1). Comparison with *D. oesophageus*, the only reptile parasite currently listed in GenBank, shared 96.7% identity (GenBank accession no. AY852269.1).

For the 28S rRNA fragment (231 bp, GenBank accession no. MT311140.1), only 1 homologous sequence from *Dracunculus* sp. HMM2018 isolate SAN-7590 sequence was available (GenBank accession no. KY990016.1) and showed an identity of 97.8%. Phylogenetic analysis using 18S rRNA data confirmed assignment to the genus *Dracunculus* and placed the specimen from Spain in a highly supported cluster containing the mammalian parasites *D. medinensis* and *D. insignis* but not the snake parasite *D. oesophageus* (Appendix Figure). Thus, morphologic and rRNA sequence data agreed in the assignment of the specimen to the genus *Dracunculus*, but no species identification was possible.

**Figure 2:**
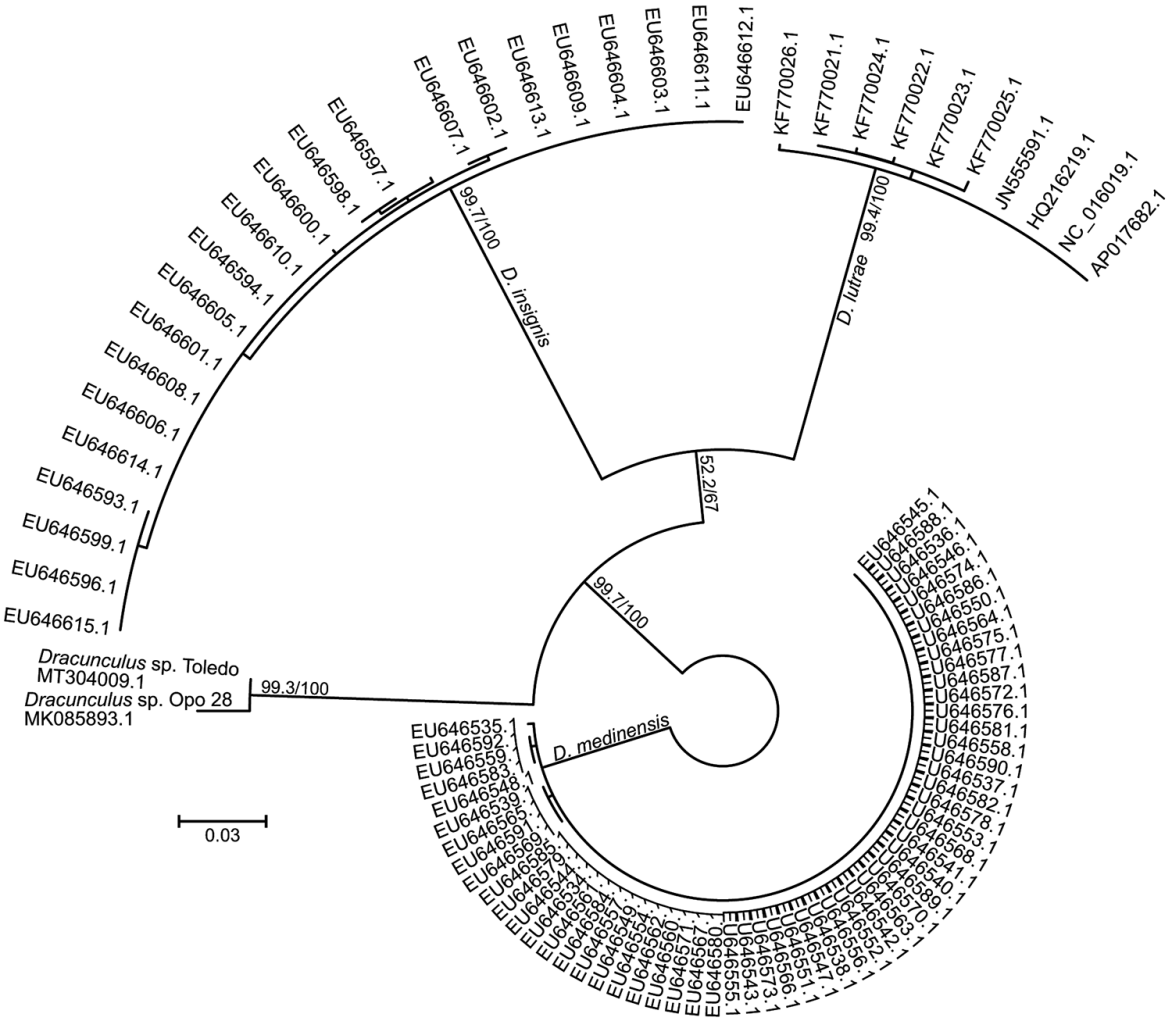
Unrooted maximum-likelihood phylogenetic tree based on *Dracunculus* spp. mitochondrial marker cytochrome oxidase C subunit I sequences from a dog in Toledo, Spain, 2018, and reference sequences. Node support values represent results of ultrafast bootstrapping before and the Shimodaira-Hasegawa approximate likelihood ratio test after the slash. Labels at end nodes represent GenBank accession numbers. Scale bar indicates substitutions per site.

The partial COI sequence (654bp, GenBank accession no. MT304009.1) showed that the nematode shared 98.3% identity with *Dracunculus* sp. Opo28 (GenBank accession no. MK085893.1), a species found in an opossum (*Didelphis virginiana*) from Georgia, USA; 92.5% with *D. insignis* (GenBank accession no. MK085896.1); 90.7% with *D. lutrae* (GenBank accession no. EU646603.1); and 89.3% identity with *D. medinensis* (GenBank accession no. AP017682.1). The phylogenetic analysis based on COI sequences positioned the nematode in a cluster together with *Dracunculus* sp. Opo28, clearly separated from the 3 other *Dracunculus* species known to infect mammals (Figure 2). The species was designated *Dracunculus* sp. Toledo.

## Conclusions

Today, the distribution of known *Dracunculus* worm species infecting mammals is limited to only a few areas endemic for *D. medinensis* worms in Africa, for *D. lutrae* worms in Canada, and for *D. insignis* worms in North America (*6*,*7*). *D. fuelleborni* worms were identified only once in an opossum in Brazil in 1934 (*8*). Canine *Dracunculus* worm infections can be caused by *D. insignis* worms in North America and *D. medinensis* worms in Africa and Asia. Rarely, canine *Dracunculus* infections have been reported from nonendemic countries such as Kazakhstan (*14*); however, because these countries have never been endemic for *D. medinensis* worms, such reports might represent infections with other species. The case in Spain represents an autochthonous mammalian case in Europe, which was considered to be free of such parasites, and manifests a knowledge gap regarding the distribution of *Dracunculus* spp. worms in mammals (*6*,*7*). Although *Dracunculus* worms had not been reported in mammals in Europe until now, unreported cases might have occurred (e.g., cases in which misidentification resulted in diagnosis of other subcutaneous nematodes, such as *Dirofilaria repens*).

The close relationship of the worm extracted in Spain to *Dracunculus* sp. Opo 28 suggests that both specimens belong to the same (or 2 closely related) species. Because *Dracunculus* sp. Opo 28 was obtained from an opossum, the possibility that both specimens represent the species *D. fuelleborni* cannot be excluded. Given that *Dracunculus* sp. Opo28 and *Dracunculus* sp. Toledo worms were found in hosts not closely related to each other as well as on different continents, North America and Europe, the species might have a wide host and geographic range. Opossums are not among the wildlife of Europe, and reservoir hosts for *Dracunculus* sp. Toledo worms need to be identified. Because *Dracunculus* sp. Opo28 and *Dracunculus* sp. Toledo worms are closely related to *D. insignis*, which frequently parasitizes raccoons, importation of this *Dracunculus* species to Europe with this invasive host species is conceivable and should be further investigated. Although the worm extracted in Spain is not *D. oesophageus*, we cannot exclude entirely the possibility that the nematode is not one of the other reptile parasites that have been described so far only morphologically. Currently, nothing is known about the presence of *Dracunculus* spp. in copepod intermediate hosts, fish and amphibian paratenic hosts, and mammalian wildlife reservoir hosts in Spain. Because the *Dracunculus* sp. Toledo worm extracted in Spain had not been reported from dogs previously, canines are probably not epidemiologically relevant hosts. Feeding on infected fish entrails was shown to be a major transmission route to dogs in Chad (*15*), and infection of the dog in Spain also likely occurred through this route. Obtaining life cycle and prevalence data will require systematic sampling of wildlife and domestic carnivores during necropsies, combined with PCR screening of copepods.

Appendix Additional information on canine *Dracunculus* nematode infection, Toledo, Spain.
