# Supplementary material for: Canine Dracunculus Nematode Infection, Toledo, Spain
Source: Emerg Infect Dis. 2020 Aug;26(8):1860–3. doi: 10.3201/eid2608.201661 (PMC7392431; doi:10.3201/eid2608.201661)
Supplement: Appendix — Additional information on canine Dracunculus nematode infection, Toledo, Spain. [file 20-1661-Techapp-s1.pdf]

Article DOI: https://doi.org/10.3201/eid2608.201661

# Canine *Dracunculus* Nematode Infection, Toledo, Spain

## Appendix

### PCRs and Sequencing

After morphological characterization, the worm was sent to Berlin for molecular analyses. PCRs targeting two nuclear loci (18S and 28S) and one mitochondrial locus (cytochrome oxidase subunit I, COI) were conducted. For the PCR each reaction contained 200 µM dNTPs, 0.5 µM of each primer pair (Appendix Table), 0.4 U Phusion Hot Start II High-fidelity DNA polymerase (Thermo Scientific) and 2 µl template DNA in 20 µl 1 × HF buffer. The PCR protocol included initial denaturation at 98°C for 30 s, 40 cycles of denaturation at 98°C for 10 s, annealing at 55°C for 30 s and elongation at 72°C for 30 s, followed by a final extension at 72°C for 5 min. PCR products were cloned and submitted for Sanger sequencing to LGC Genomics (Berlin).

### Phylogenetic Analyses

The CO1 DNA sequences were aligned codon-wise using MUSCLE (*1*) as implemented in Mega7 (*2*). The alignment of rRNA sequences was performed with MAFFT using the command line command line: mafft -qinsi–maxiterate 2–reorder input. The optimal substitution models and the phylogenetic tree were determined on the IQTREE web server (*3*) applying the Modelfinder (*4*) and IQTREE (*5*) softwares, respectively. Modelfinder was run using autoselect of the optimal substitution model also considering FreeRate heterogeneity models. Both, ultrafast bootstrapping (*6*) and Shimodaira–Hasegawa approximate likelihood ratio tests (SH-aLRTs) (*7*) were conducted with 1000 replicates. The final command line was: path_to_iqtree -s iqtree_Alignment.phy -st DNA -m TESTNEW -bb 1000 -alrt 1000. Trees were visualized using FigTree v1.4.4.

**References**


1. Edgar RC. MUSCLE: multiple sequence alignment with high accuracy and high throughput. Nucleic Acids Res. 2004;32:1792–7. PubMed https://doi.org/10.1093/nar/gkh340

2. Kumar S, Stecher G, Tamura K. MEGA7: Molecular Evolutionary Genetics Analysis Version 7.0 for bigger datasets. Mol Biol Evol. 2016;33:1870–4. PubMed https://doi.org/10.1093/molbev/msw054

3. Trifinopoulos J, Nguyen LT, von Haeseler A, Minh BQ. W-IQ-TREE: a fast online phylogenetic tool for maximum likelihood analysis. Nucleic Acids Res. 2016;44(W1):W232-5. PubMed https://doi.org/10.1093/nar/gkw256

4. Kalyaanamoorthy S, Minh BQ, Wong TKF, von Haeseler A, Jermiin LS. ModelFinder: fast model selection for accurate phylogenetic estimates. Nat Methods. 2017;14:587–9. PubMed https://doi.org/10.1038/nmeth.4285

5. Nguyen LT, Schmidt HA, von Haeseler A, Minh BQ. IQ-TREE: a fast and effective stochastic algorithm for estimating maximum-likelihood phylogenies. Mol Biol Evol. 2015;32:268–74. PubMed https://doi.org/10.1093/molbev/msu300

6. Hoang DT, Chernomor O, von Haeseler A, Minh BQ, Vinh LS. UFBoot2: improving the ultrafast bootstrap approximation. Mol Biol Evol. 2018;35:518–22. PubMed https://doi.org/10.1093/molbev/msx281

7. Guindon S, Dufayard JF, Lefort V, Anisimova M, Hordijk W, Gascuel O. New algorithms and methods to estimate maximum-likelihood phylogenies: assessing the performance of PhyML 3.0. Syst Biol. 2010;59:307–21. PubMed https://doi.org/10.1093/sysbio/syq010

8. Duscher GG, Harl J, Fuehrer HP. Evidence of *Troglotrema acutum* and *Skrjabingylus* spp. coinfection in a polecat from Lower Austria. Helminthologia. 2015;52:63–6. https://doi.org/10.1515/helmin-2015-0011

9. Demeler J, Ramünke S, Wolken S, Ianiello D, Rinaldi L, Gahutu JB, et al. Discrimination of gastrointestinal nematode eggs from crude fecal egg preparations by inhibitor-resistant conventional and real-time PCR. PLoS One. 2013;8:e61285. PubMed https://doi.org/10.1371/journal.pone.0061285

10. Blaxter ML, De Ley P, Garey JR, Liu LX, Scheldeman P, Vierstraete A, et al. A molecular evolutionary framework for the phylum Nematoda. Nature. 1998;392:71–5. PubMed https://doi.org/10.1038/32160

**Appendix Table.** PCR primers used to amplify *Dracunculus* spp. DNA fragments

| Primer name | Target | Sequence | Reference |
|---|---|---|---|
| COI_Nema_Fw | Cytochrome c oxidase subunit 1 | GAAAGTTCTAATCATAARGATATTGG | (*8*) |
| COI_Nema_Rv | Cytochrome c oxidase subunit 1 | ACCTCAGGATGACCAAAAAAYCAA | (*8*) |
| Nematode-18sfor | 18S rDNA | AACTGCGAACGGCTCATTAGAGCAGATGT | (*9*) |
| SSU_R26 | 18S rDNA | CATTCTTGGCAAATGCTTTCG | (*10*) |
| Nematode-28sfor | 28S rDNA | GGCGAGTGAACGGGGAGAAGCCCAGCGCTGA | (*9*) |
| Nematode-28srev | 28S rDNA | TTTCCTTCACAGTACTTGTTTGCTATCGAAT | (*9*) |

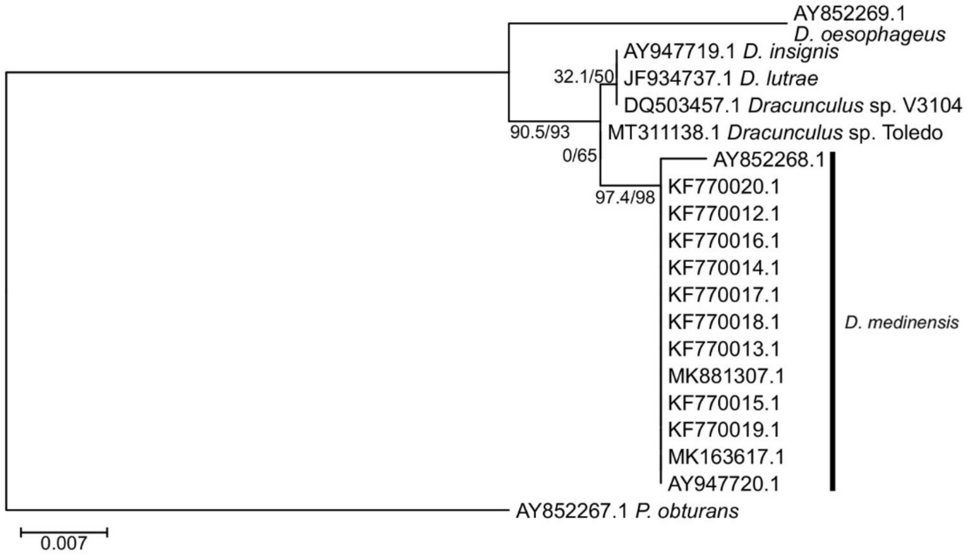


**Appendix Figure.** Maximum-likelihood phylogenetic tree of *Dracunculus* spp. sequences available from GenBank. The tree was rooted using a sequence from the nematode *Philometra obturans* (Spirurida, Dracunculoidea, Philometridae) as outgroup. The scale bar represents 0.007 substitutions per site. Node support values before and behind the slash provide the results of ultrafast bootstrapping and the Shimodaira-Hasegawa likelihood ratio test. GenBank accession numbers were used an end node labels.
